# COVID-19 pandemic effects on neonatal inpatient admissions and mortality: interrupted time series analysis of facilities implementing NEST360 in Kenya, Malawi, Nigeria, and Tanzania

**DOI:** 10.1186/s12887-024-04873-1

**Published:** 2024-07-08

**Authors:** Lucas Malla, Eric O. Ohuma, Josephine Shabani, Samuel Ngwala, Olabisi Dosunmu, John Wainaina, Jalemba Aluvaala, Irabi Kassim, James H. Cross, Nahya Salim, Evelyn Zimba, Chinyere Ezeaka, Rebecca E. Penzias, David Gathara, Robert Tillya, Msandeni Chiume, Opeyemi Odedere, Norman Lufesi, Kondwani Kawaza, Grace Irimu, Olukemi Tongo, Sarah Murless-Collins, Christine Bohne, Rebecca Richards-Kortum, Maria Oden, Joy E. Lawn

**Affiliations:** 1 Maternal, Adolescent, Reproductive & Child Health (MARCH) Centre, London School of Hygiene & Tropical Medicine, London, UK; 2 Health Systems Impact Evaluation and Policy Department, Ifakara Health Institute, Dar Es Salaam, Tanzania; 3 Research Support Center, School of Public Health and Family Medicine, Kamuzu University of Health Sciences, Blantyre, Malawi; 4 APIN Public Health Initiatives, Abuja, Nigeria; 5 Kenya Medical Research Institute-Wellcome Trust, Nairobi, Kenya; 6 Department of Paediatrics and Child Health, Muhimbili University of Health and Allied Sciences, Dar Es Salaam, Tanzania; 7 Department of Health Systems, Impact Evaluation and Policy, Ifakara Health Institute, Dar Es Salaam, Tanzania; 8 Rice360 Institute for Global Health Technologies, Rice University, Houston, TX USA; 9 Department of Paediatrics, College of Medicine, University of Lagos, Lagos, Nigeria; 10 Department of Paediatrics, Kamuzu University of Health Sciences (Formerly College of Medicine, University of Malawi), Blantyre, Malawi; 11 Department of Curative and Medical Rehabilitation, Ministry of Health, Lilongwe, Malawi; 12 Department of Paediatrics and Child Health, University of Nairobi, Nairobi, Kenya; 13 Department of Paediatrics, College of Medicine, University of Ibadan, Ibadan, Nigeria

**Keywords:** Newborn, Low- and middle-income countries, Inpatient care, COVID, Interrupted time series, Neonatal mortality

## Abstract

**Background:**

The emergence of COVID-19 precipitated containment policies (e.g., lockdowns, school closures, etc.). These policies disrupted healthcare, potentially eroding gains for Sustainable Development Goals including for neonatal mortality. Our analysis aimed to evaluate indirect effects of COVID-19 containment policies on neonatal admissions and mortality in 67 neonatal units across Kenya, Malawi, Nigeria, and Tanzania between January 2019 and December 2021.

**Methods:**

The Oxford Stringency Index was applied to quantify COVID-19 policy stringency over time for Kenya, Malawi, Nigeria, and Tanzania. Stringency increased markedly between March and April 2020 for these four countries (although less so in Tanzania), therefore defining the point of interruption. We used March as the primary interruption month, with April for sensitivity analysis. Additional sensitivity analysis excluded data for March and April 2020, modelled the index as a continuous exposure, and examined models for each country. To evaluate changes in neonatal admissions and mortality based on this interruption period, a mixed effects segmented regression was applied. The unit of analysis was the neonatal unit (*n* = 67), with a total of 266,741 neonatal admissions (January 2019 to December 2021).

**Results:**

Admission to neonatal units decreased by 15% overall from February to March 2020, with half of the 67 neonatal units showing a decline in admissions. Of the 34 neonatal units with a decline in admissions, 19 (28%) had a significant decrease of ≥ 20%. The month-to-month decrease in admissions was approximately 2% on average from March 2020 to December 2021. Despite the decline in admissions, we found no significant changes in overall inpatient neonatal mortality. The three sensitivity analyses provided consistent findings.

**Conclusion:**

COVID-19 containment measures had an impact on neonatal admissions, but no significant change in overall inpatient neonatal mortality was detected. Additional qualitative research in these facilities has explored possible reasons. Strengthening healthcare systems to endure unexpected events, such as pandemics, is critical in continuing progress towards achieving Sustainable Development Goals, including reducing neonatal deaths to less than 12 per 1000 live births by 2030.

**Supplementary Information:**

The online version contains supplementary material available at 10.1186/s12887-024-04873-1.

## Key findings

1. What was known?
  The COVID-19 pandemic disrupted provision, and utilisation of health services. However, few publications have reported primary data from multi-country settings evaluating neonatal care and outcomes associated with this disruption.
2. What was done that is new?
  Data from 67 neonatal units constituting 266,741 admission records from January 2019 to December 2021 across Malawi, Kenya, Nigeria, and Tanzania were analysed to evaluate changes in neonatal admissions, case mix and mortality.
  The Oxford Stringency Index was used to quantify policy shifts across the four countries to determine interruption time point for an Interrupted Time Series analysis. A mixed effects segmented regression was applied.
3. What was found?
  Implementation of policies appeared more stringent from April 2020. Overall admissions into the NEST360 neonatal units were reduced by 15%, with an average monthly reduction across all the facilities of about 2% after March 2020.
  Half of the units showed either significant (*n* = 19) or borderline (*n* = 15) step reductions. Further, a quarter of the units showed a significant month-to-month decline in admissions by at least 2%.
  There was no measurable change detected in pooled mortality, although some facilities reported decreases and increases in neonatal mortality.
4. What next?
  Currently, 63 countries are off track to achieve the Sustainable Development Goal 3.2 of reducing neonatal deaths to 12 per 1000 live births by 2030. These efforts may be further derailed by unexpected disruptions to healthcare systems.
  To mitigate future risks, it is essential to enhance the capacity of health systems to respond quickly and effectively to pandemics, making them more resilient to shocks.

## Background

Progress has been made in improving child health and survival over the past two decades, with global under-five child mortality reduced by more than half, between 1990 and 2021 [1]. Yet there were an estimated 2.3 million neonatal deaths globally in 2021 [2], representing 47% of under-five deaths, with slower progress made in reducing mortality during the neonatal period (28 days after birth). Reducing neonatal mortality, especially amongst vulnerable newborns [3], is imperative to achieving the Sustainable Development Goal (SDG) target 3.2 for every country to reduce preventable newborn deaths to at least 12 per 1,000 live births and under-five child deaths to at least 25 per 1,000 live births by 2030 [4].

The COVID-19 pandemic resulted in major public health shifts in all countries, causing millions of deaths worldwide [5]. Governments imposed containment measures such as isolation of infected persons, quarantines, and lockdowns. These measures disrupted provision and utilisation of health services [6, 7], indirectly affecting outpatient and emergency care, especially for the most vulnerable [8].

Newborns and mothers are particularly vulnerable users of any health system [9]. Disruptions of the health system can negatively impact health outcomes for mothers and their newborns, especially those born premature, too small or those who become sick [8]. To achieve SDG target 3.2 of neonatal mortality target of < 12 deaths per 1000 live births, high-quality small and sick newborn care (SSNC) is critical [10].

The Newborn Essential Solutions and Technologies (NEST360) Alliance was formed to support African governments in their commitment to reducing inpatient neonatal deaths through implementation of a sustainable health system strengthening package including innovative devices, training, data systems, and quality improvement with mentorship. The NEST360 Alliance aims to improve the quality of SSNC in 67 facilities across four countries: Kenya, Malawi, Nigeria, and Tanzania.

Whilst disruptions associated with the COVID-19 pandemic have been shown to indirectly affect many aspects of healthcare [8], there is little published multi-country, primary data on the impact of COVID-19 disruption on neonatal admissions and mortality. Some published studies were based on modelling, or surveys, and those with primary data have also shown varied findings [8, 11–15]. This paper is part of a supplement reporting learnings with the NEST360 Alliance.

## Objectives

Our aim was to evaluate the indirect impact of COVID-19 containment measures on inpatient neonatal admission and mortality. Specifically, we:

1. Compared stringency of COVID-19 policies in four countries implementing with the NEST360 Alliance (Kenya, Malawi, Nigeria, and Tanzania).
2. Quantified the indirect impact of COVID-19 on neonatal admissions, case-mix, and inpatient neonatal mortality in 67 neonatal units implementing with NEST360 between January 2019 and December 2021.

## Methods

### Study setting

The 67 facilities affiliated with NEST360 comprised of 69 neonatal units, as some facilities in Nigeria had geographically separated inborn and outborn units. Two units in Malawi did not have neonatal inpatient data and were therefore excluded, resulting in 67 neonatal units as the final total for analysis. Based on the World Health Organization (WHO) level of newborn care [16, 17] and country classifications, of the 67 neonatal units, 33 are District level (31 in Malawi), 24 are Secondary (10 in Kenya), and 10 are Tertiary (5 in Nigeria). We examined data for the period January 2019 to December 2021.

### Study design

We applied an Interrupted Time Series (ITS) design to assess the effect of COVID-19 containment measures on neonatal admissions and mortality. ITS evaluates change by two metrics – "step" and "slope" change parameters [18]. Estimation of step and slope change requires prior definition in ITS design. We used the Oxford Stringency Index, which defines the level of stringency of policies in various countries and was computed by the Blavatnik School of Government (University of Oxford) [19].

### Admission and mortality data

Newborn admission and mortality data were obtained from the Neonatal Inpatient Dataset (NID). The NEST360 Alliance, with four country governments, co-developed the NID intended to enable and track scale-up of high-quality WHO level-2 SSNC with respiratory support in hospitals [20]. The NID consists of 60 core variables organised into six modules: (1) birth details/maternal history; (2) admission details/identifiers; (3) clinical complications/observations; (4) interventions/investigations; (5) discharge outcomes; and (6) diagnosis/cause-of-death.

In the neonatal units, the data collection takes place post-discharge of the neonates and is carried out by trained data officers. They adhere to well-defined standard operating procedures established by the research team. The patient file, serving as the formal documentation of the clinical condition and management, plays a crucial role in this process. The file contains admission information, treatment sheets, discharge summary forms, laboratory reports, and general clinical notes. In neonatal units where Electronic Health Records (EHRs) are implemented, we have established a linkage between the NID and the EHRs. This integration facilitates seamless data abstraction, contributing to enhanced data quality and efficiency in the overall process.

We developed and utilised a novel approach to account for underreporting of data for the smallest babies in neonatal admissions and mortality. Number of admissions and deaths for newborns with either low birthweight (i.e., < 2500g) or gestational age (i.e., < 28 weeks) tend to be underreported in both low- and high-income settings, and especially in routine data systems [21–24]. Some factors influencing underreporting include: (i) health worker misunderstanding of registration guidance (e.g., applying stillbirth registration threshold of 1000g to neonatal deaths) [25]; (ii) physician perception of viability of the smallest babies, especially considering increased survival of micro-preemies globally [26], and (iii) negative implications associated with reporting (e.g., penalisation of hospitals with higher mortality rates, effort required for more data collection, etc.). The potential impact of COVID-19 disruption is likely to be underestimated if underreporting is not accounted for in the analysis.

The NID was examined for evidence of underreporting for the lowest birthweight categories, using distributions of admissions by birthweight category and the corresponding birthweight-specific mortality (BWSM) curves, also considering time trends. We identified evidence of underreporting, based on implausible birthweight specific mortality curves especially of neonates < 1000g birthweight. A criterion of Neonatal Mortality Rate (NMR) ≥ 700 deaths per 1000 live births for neonates weighing < 1000g was used. This cut-off was considered conservative since mortality in this group without full intensive care was reported to be at least 800 per 1000 live births [24, 27]. Hence using selected neonatal units meeting the criteria for more plausible birthweight-specific mortality for < 1000g, we developed standardised curves. These curves were then applied to the rest of the dataset to adjust for the undercounting of both deaths and admissions by facility (Additional File 1).

### Statistical analyses

The neonatal unit was the unit of analysis, and time was defined as calendar months. For the ITS design, we defined a *step change* as the average modelled change observed between the month when COVID-19 policy stringency increased (i.e., March 2020) as the point of interruption compared to the preceding month (i.e., February 2020). We defined a *slope change* as the modelled average month-to-month changes observed in the time-series data before the interruption period (January 2019 to February 2020) compared to after the interruption period (i.e., April 2020 to December 2021). Data from January 2019–February 2020 (pre-interruption) was classified as "pre-COVID," and from April 2020–December 2021 as "during COVID". Our ITS impact model evaluated and quantified both immediate step change and month-to-month effects as slope change [18].

The analysis examined overall and unit-level effects, which were estimated using segmented mixed-effects regression in the Frequentist framework, with the primary point of interruption being March 2020. The models for admissions and NMRs data were of the following general form (with differences reflected in the distributions and link functions applied):

1 $${y}_{ij}= {\beta }_{0j}+ {\beta }_{1j}{Time}_{ij}+ {\beta }_{2j}{Phase}_{ij}+ {\beta }_{3j}{Time \,after}_{ij}+{\varvec{\upbeta}}\mathbf{X}+ {\mathcal{e}}_{ij}$$

2 $$\begin{array}{c}{\beta }_{0j}= {\mu }_{00}+ {r}_{0j}\\ {\beta }_{1j}= {\mu }_{10}+ {r}_{1j}\\ \begin{array}{c}{\beta }_{2j}= {\mu }_{20}+ {r}_{2j}\\ {\beta }_{3j}= {\mu }_{30}+ {r}_{3j}\end{array}\end{array}$$

where:

- ${y}_{ij}$ is the adjusted number of either admissions or neonatal mortality rate at *i*^th^ month for the *j*^th^ neonatal unit.
- ${\beta }_{0j}$ denotes pre-interruption intercept for the *j*^th^ neonatal unit.
- ${\beta }_{1j}$ denotes pre-interruption slope for the *j*^th^ neonatal unit.
- ${\beta }_{2j}$ denotes the step change for the *j*^th^ neonatal unit after interruption.
- ${\beta }_{3j}$ denotes slope change for the *j*^th^ neonatal unit after interruption.
- ${\mu }_{00}$ denotes pre-interruption average intercept
- ${\mu }_{01}$ denotes pre-interruption average slope.
- ${\mu }_{02}$ denotes average step change
- ${\mu }_{03}$ denotes average slope change
- ${r}_{0j}$, ${r}_{1j}$, ${r}_{2j}$ and ${r}_{3j}$ are neonatal unit-specific random effects for pre-interruption intercept, pre-interruption slope, step change after interruption, and slope change after interruption, respectively.
- ${\varvec{\upbeta}}$ denotes a matrix of corresponding parameters for fixed effects including country and non-linear functions of time modelled using harmonic terms [28].

To account for overdispersion, we modelled the monthly number of admissions at the neonatal unit level using a negative binomial distribution with a log link function [29]. NMRs were expressed as proportions and modelled using a beta distribution with a logit link function to restrict adjusted and fitted values to plausible ranges between 0 and 1 [30]. For all models, we smoothed trends using three monthly moving averages. The likelihood ratio test was used to evaluate the importance of including harmonic terms in mean structures and equal tailed uncertainty intervals for random effects were computed using Wald z-distribution approximation. Further, the analysis compared the performance of unstructured and autoregressive variance covariance structures based on how each optimised the likelihood function (Additional File 1 – eTable 1).

Four sensitivity analyses were conducted to assess the robustness of the ITS modelling of admissions and mortality. First, April 2020 was examined as an interruption time point and segmented mixed effects models re-fitted. Second, data for March and April 2020 were excluded to evaluate their impact on the modelling results, followed by re-fitting of the models using the remaining data. Third, the Oxford Stringency Index was treated as a continuous exposure, with changes in admissions and mortality modelled using an interaction term between the index and time in months. Lastly, the analysis was stratified and segmented regression models fitted to the data for each country.

An exploratory sub-group analysis was performed to investigate any changes in admission by primary diagnosis and referral patterns (i.e., whether neonates were born within the hospital or referred). This subgroup analysis was performed using pooled data and did not include fitting models to assess the statistical significance of changes in primary diagnosis and referral patterns. All the analyses were conducted in R version 4.3.2 [31].

## Results

A total of 266,741 newborn records from 67 neonatal units were available from January 2019 to December 2021. Most admissions were in Malawi (*N* = 36, *n* = 126,578 (47.5%)), followed by Kenya (*N* = 13, *n* = 73,608 (28%)), Tanzania (*N* = 7, *n* = 53,558 (20%)), and Nigeria (*N* = 11, *n* = 12,997 (5%)) (Table 1).

**Table 1:** NEST360 neonatal inpatient data by country (up to December 2021)

Country	Number of facilities	Number of neonatal units with baseline data	Total Admission records	January 2019 – March 2020 (Pre-COVID)	April 2020 – Dec 2021 (During COVID)
Malawi	38	36	126,578	53,206	73,372
Kenya	13	13	73,608	31,850	41,758
Tanzania	7	7	53,558	22,991	30,567
Nigeria	7	11	12,997	5840	7157
**Total**	**65**	**67**	**266,741**	**113,887**	**152,854**

Two facilities in Malawi are new and, therefore, do not currently have any data. Nigeria has seven facilities with four of them split into separate inborn and outborn neonatal units. All analyses have been undertaken with the neonatal unit as the unit of analysis; therefore, analysis is based on 67 neonatal units

### Interruption time-point(s) based on the Oxford stringency index

**Fig. 1:**
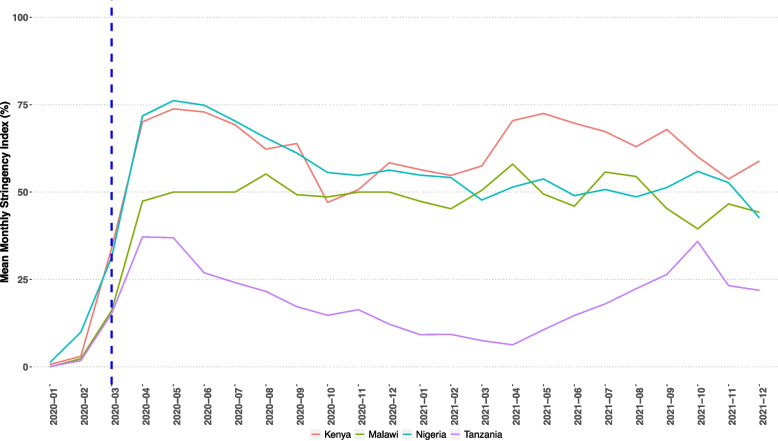
The Oxford Stringency Index summarised by month and the four NEST360 countries

Oxford Stringency Index scores were summarised for each of the four countries implementing with NEST360. Variations were observed among countries and over time, with policies appearing stricter in Kenya, Malawi, and Nigeria and less so in Tanzania. Implementation of policies started in March 2020, becoming more stringent by April 2020 (Fig. 1). Data from January 2019–March 2020 accounted for 43% (*n* = 113,887) of neonatal records (Table 1).

### Changes in admission and NMR patterns using March 2020 as interruption time-point

Additional File 1 – eFigures 4a and 4b show good fit for March 2020 interruption models (and for April 2020 interruption), which is demonstrated using data for selected neonatal units from each of the four countries. Fitted admission and mortality trends for all neonatal units using the March 2020 interruption models are presented in Additional File 1 – eFigures 5a and b. Similar trends were obtained using the April 2020 interruption models and are presented in Additional File 1 – eFigures 6a and b.

### Changes in admission trends

**Fig. 2:**
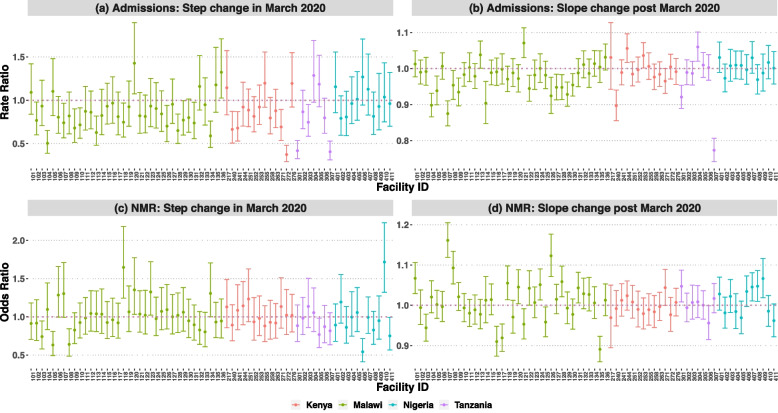
Facility level admission and NMR step and slope change estimates for March 2020 interruption time-point with 95% CI for NMR**.** Abbreviations**:** NMR; neonatal mortality rate, CI; confidence interval

In March 2020, there was a significant 15% (Rate Ratio = 0.85; 95% Confidence Interval (CI): 0.78–0.93) decline in overall number of admissions to all neonatal units compared to February 2020 (Table 2). Half of the neonatal units (*N* = 34/67) reflected this decrease, with 19 of them experiencing a significant reduction by ≥ 20%. The remaining 15 demonstrated borderline effects (Fig. 2a). In each country, among the 34 neonatal units, 5 of 7 units in Tanzania, 8 of 13 units in Kenya, 3 of 11 units in Nigeria, and 18 of 36 units in Malawi showed either significant or borderline step reductions in admissions between February and March 2020.

**Table 2:** Model estimates for March and April 2020 interruption

	**March 2020 Interruption**		**April 2020 Interruption**		**March–April Washout**	
	**Admissions**	**NMR**	**Admissions**	**NMR**	**Admissions**	**NMR**
	**Rate Ratio** **[95% CI]**	**Odds Ratio** **[95% CI]**	**Rate Ratio** **[95% CI]**	**Odds Ratio** **[95% CI]**	**Rate Ratio** **[95% CI]**	**Odds Ratio** **[95% CI]**
Step change	**0.85 [ 0.78** – **0.93]**	0.95 [ 0.89 – 1.03]	**0.82 [0.75 – 0.89]**	0.98 [0.91 – 1.06]	**0.79 [0.74 – 0.83)**	1.00 [0.64 – 1.58]
Slope change	0.98 [ 0.97 – 0.99]	1.00 [ 0.99 – 1.01]	0.98 [0.97 – 1.00]	1.00 [ 0.99 – 1.01]	0.98 [0.97 – 0.99]	1.00 [1.00 – 1.06]

The segmented models adjusted for country as a fixed effect variable as well as Fourier terms to account for non-linearity in the trends

*NMR* neonatal mortality rate, *CI* confidence interval

From March 2020 onwards, the overall month-to-month (slope) decrease in admissions remained at approximately 2% (Rate Ratio = 0.98; 95% CI: 0.97–0.99) (Table 2). Of the 67 neonatal units, 15 (12 of 36 units in Malawi, 2 of 7 units in Tanzania and 1 of 13 units in Kenya) showed a significant month-to-month decrease in admissions by ≥ 2%. In contrast, four neonatal units (2 of 36 units in Malawi, 1 of 7 units in Tanzania, and 1 of 13 units in Kenya) had a significant month-to-month increase in admissions by ≥ 3%. No measurable month-to-month changes were observed in neonatal units in Nigeria (Fig. 2b). Similar overall and neonatal unit step and slope changes were observed for the April 2020 interruption models (see Additional File 1 – eFigure 7). Additionally, the analysis that excluded data for March and April 2020 also showed approximately similar findings (Table 2).

### Changes in neonatal mortality trends

No measurable change in overall neonatal mortality was observed between February and March 2020 (Odds Ratio = 0.95; 95% CI: 0.89–1.03) (Table 2). However, specific neonatal units experienced significant step changes in neonatal mortality. Of the 67 neonatal units, 6 had a significant step increase (5 of 36 units in Malawi and 1 of 11 units in Nigeria) in neonatal mortality by ≥ 30%, while 6 units (3 of 36 units in Malawi, 1 of 7 units in Tanzania, and 2 of 11 units in Nigeria) had a step decrease by ≥ 25% (Fig. 2c).

Further analysis of month-to-month changes across all units showed no slope change in neonatal mortality (Odds Ratio = 1.00; 95% CI: 0.99–1.01) (Table 2). A significant month-to-month increase in neonatal mortality by ≥ 5% was observed in 13 units (9 of 36 units in Malawi and 4 of 11 units in Nigeria). In contrast, 7 of 36 neonatal units in Malawi reported a significant month-to-month decrease in NMR by ≥ 4% (Fig. 2d).

### Changes in neonatal admission case mix

When analysing trends of neonates admitted with specific conditions, there were minimal changes in trends for neonates primarily admitted due to congenital malformations, intrapartum-related illnesses, and jaundice. Neonates admitted for prematurity showed a slight increase over time (from ~ 25% in January 2019 to ~ 30% in December 2021), while those admitted for infections showed a decrease, particularly between April (~ 25%) and August 2020 (~ 12%) (Additional File 1 – Fig. 9a). From March 2020 onwards, there was a decrease in the number of neonates born both within and outside the facilities. The decline was slightly more pronounced for neonates born within the facilities compared to those born outside (Additional File 1 – Fig. 9b).

### Analysis using Oxford Stringency Index as a continuous exposure

The interaction term between time in months and the Oxford Stringency Index showed an overall significant reduction in admission numbers over time. However, a similar analysis did not show any significant change in overall mortality (Additional File 1 – eTable 2). See the fitted trends for admissions and NMR in Additional File 1 – Figs. 8a and b.

### Stratified analysis for each country with March as an interruption time-point

In the country-specific models, 29 out of 67 neonatal units showed either borderline or significant reductions in admission numbers compared to 34 out of 67 we obtained in the pooled analysis for all countries (see Additional File 1 – eTable 3 and 4). The difference of seven units were mostly in Malawi (six of the seven units) and one in Tanzania. The six neonatal units in Malawi are small; hence, the differences could potentially be attributed to reduced sample sizes.

## Discussion

This study used a large dataset of 266,741 neonatal admissions to examine indirect effects of COVID-19 containment measures on admissions and mortality trends in 67 neonatal units implementing with NEST360 in Kenya, Malawi, Nigeria, and Tanzania. We used the Oxford Stringency Index to define the month of interruption and found a 15% decline in neonatal admissions overall between February and March 2020 and an average month-to-month reduction in admissions by 2% between March 2020 and December 2021. Despite the decline in admissions, we found no significant changes in overall neonatal mortality (Odds Ratio = 0.95; 95% CI: 0.89–1.03). However, results varied across individual neonatal units, i.e., between February and March 2020, six neonatal units showed a significant increase in neonatal mortality by ≥ 30%, whereas six neonatal units showed a decrease by ≥ 25%. Similarly, between March 2020 and December 2021, a significant month-to-month increase (≥ 5%) in neonatal mortality was observed in 13 units compared to seven units that reported a significant decrease.

Our analysis showed a substantial effect on neonatal admissions, aligning with findings from other studies evaluating neonatal admissions during the pandemic [8, 11, 32]. These results are unsurprising as COVID-19 containment measures caused disruption of health service delivery and demand across various levels of healthcare, including prenatal, maternal, and paediatric care [33]. COVID-19 reported effects on neonatal mortality have been mixed. For example, some studies found no significant mortality change, whereas others reported increases [8, 11–15, 34]. Specifically, studies in Zimbabwe, Malawi, Ireland and Botswana found no impact of COVID-19 on neonatal mortality [12, 13, 34], whereas a study conducted in Nepal reported a significant increase in institutional neonatal mortality, from 13 to 40 deaths per 1000 live births, noting that this was on labour ward [11]. A study in Turkey found little change [25], while two studies in Uganda reported a relative increase in neonatal mortality by 25–30% [8, 14]. Studies which reported increased mortality during COVID-19 might be related to reprioritisation of health resources, delayed presentation to the facilities, late diagnosis, and partial immunisation coverage, among other reasons [8, 14].

Based on this quantitative analysis, the NEST360 Alliance conducted a separate qualitative study in these neonatal units to better understand barriers and protective factors, as well as learn from pivots during the COVID-19 pandemic [35]. This qualitative study suggested several ways that SSNC was protected during the pandemic. One theme that emerged was around COVID-specific opportunities from the pandemic including more focus on infection control measures to prevent the spread of COVID-19, which may also have helped reduce other infections in the units. Another protective mechanism was utilising technology like telemedicine to facilitate patient-provider communication and monitor patient health. Additionally, access to oxygen and routine maintenance of medical devices received new investment. This qualitative study also provided valuable insights into the gaps for SSNC, which is still new on the global health agenda, and the need for increased investment in infrastructure and workers to enable more resilience to future shocks.

Our ITS study has strengths and limitations. One obvious strength is the large dataset of more than a quarter of a million records from 67 neonatal care units across four countries, being the largest primary neonatal analyses published to date on the indirect impact of COVID-19. The rigour of the analyses included robust ITS modelling with three different sensitivity analyses approaches, still resulting in consistent findings. Limitations include reliance on the Oxford Stringency Index which was based on national policy and contingency measures that were put in place in response to COVID-19 and may not be reflective of what actually happened in practice. Additionally, we were not able to consistently account for sub-national variation in implementation of COVID-19 containment measures, which would be particularly relevant in large, decentralised countries such as Nigeria. The use of routine facility-reported data may miss admissions and deaths, especially of the smallest neonates, and even though we attempted to adjust for underreporting, it is difficult to judge how representative the adjusted estimates are from the “truth” in the absence of a gold standard. The NEST360 Alliance is working with these facilities and the government to improve neonatal data quality in routine national systems.

## Conclusion

We found an overall decrease in neonatal admissions associated with COVID-19 containment measures, but without measurable change in newborn mortality across 67 neonatal units. There is a need to protect all care, including newborn care, by allocating sufficient resources [36]. Healthcare systems need practical approaches to resilience, including policies that can adapt to rapidly changing circumstances [37, 38]. The linked qualitative research found remarkable examples of local leadership by healthcare providers, government, and partners. Importantly many stakeholders need to be more aware of the vulnerability of newborns and their families at times of crises and use their voices and resources to protect them [39].

## Supplementary Information

Additional file 1. The file highlights the adjustment approach in detail and also provides additional results referenced in the main paper.

Additional file 2. Ethics approval of Institutional Review Boards.

## Data Availability

All partners in the NEST360 alliance collaborated to create and sign data sharing and transfer agreements. The dataset from this study will be accessible upon request, pending approval from the NEST360 learning network and collaborating parties.

## Abbreviations

COVID-19: Coronavirus Infectious Disease Pandemic
CI: Confidence Interval
ITS: Interrupted Time Series
NEST360: Newborn Essential Solutions and Technologies
NID: Neonatal Inpatient Dataset
NMR: Neonatal Mortality Rate
SDGs: Sustainable Development Goals
SSNC: Small and Sick Newborn Care
WHO: World Health Organization
