# Supplementary material for: COVID-19 pandemic effects on neonatal inpatient admissions and mortality: interrupted time series analysis of facilities implementing NEST360 in Kenya, Malawi, Nigeria, and Tanzania
Source: BMC Pediatr. 2024 Jul 8;23(Suppl 2):657. doi: 10.1186/s12887-024-04873-1 (PMC11232189; doi:10.1186/s12887-024-04873-1)
Supplement: Supplementary file 1 — Additional file 1. The file highlights the adjustment approach in detail and also provides additional results referenced in the main paper. [file 12887_2024_4873_MOESM1_ESM.docx]

**Supplementary File 1**

**The indirect impact of COVID-19 pandemic on neonatal inpatient admissions and mortality in NEST360 implementing facilities in four African countries: An interrupted time series analysis**

This supplementary material presents a comprehensive overview of the methodology employed in correcting the under-reporting of hospital admissions and deaths. Moreover, it furnishes supplementary data for the comparison between unstructured and autoregressive variance covariance structures, results for March and April 2020 interruption point models, the analysis results obtained by considering the Oxford Stringency Index as a continuous exposure, along with results for country specific analysis. Additionally, it includes the diagnostic evaluation of the model fit.

**Summary**

Undercounting was apparent for 33 units that reported fewer than 700 deaths per 1000 live births for neonates weighing less than 1000grams (e**Figure 1 a**). Unadjusted admissions and NMR trends also showed evidence of undercounting as some neonatal units reported fewer admissions and zero deaths over an extended time period (**eFigure 1b and eFigure 1c**). A subset of 34 neonatal units with plausible data was chosen for the development of admission and neonatal mortality rate (NMR) adjustment functions (**eFigure 1d**). The goodness of fit for the adjustment functions was evaluated by comparing the observed and predicted data for the 34 selected neonatal units, and all were considered satisfactory as there were substantial overlaps between observed (dots) and fitted (blue lines) both for admissions and mortality data (**eFigures 2 a and b**). The estimated adjustment functions were then used to adjust for underreporting and data gaps in the remaining facilities data. **eFigures 3 a and b** represent the adjusted and three-monthly moving average smoothed trends for admissions and mortality data.

**Steps for adjustment approach**

**Step 1**: Examining evidence of underreporting in NEST360 neonatal units

The NEST360 mortality dataset was initially analysed to determine the presence of under-reporting in the lowest birth weight categories. This was accomplished through the examination of the distributions of hospital admissions based on birth weight categories and the corresponding birth weight-specific mortality (BWSM) curves. Underreporting was noted for both admission and mortality data across all the birthweight categories. In particular, some units reported zero deaths and unrealistic NMR values, especially for babies weighing less than 1000g (as shown in eFigure 1a). Further evidence of underreporting was assessed by analysing unadjusted admission and mortality trends. eFigures 1b and c showed evidence of undercounting, as some neonatal units (mostly in Malawi and Nigeria) reported fewer admissions and zero deaths over an extended period of time.


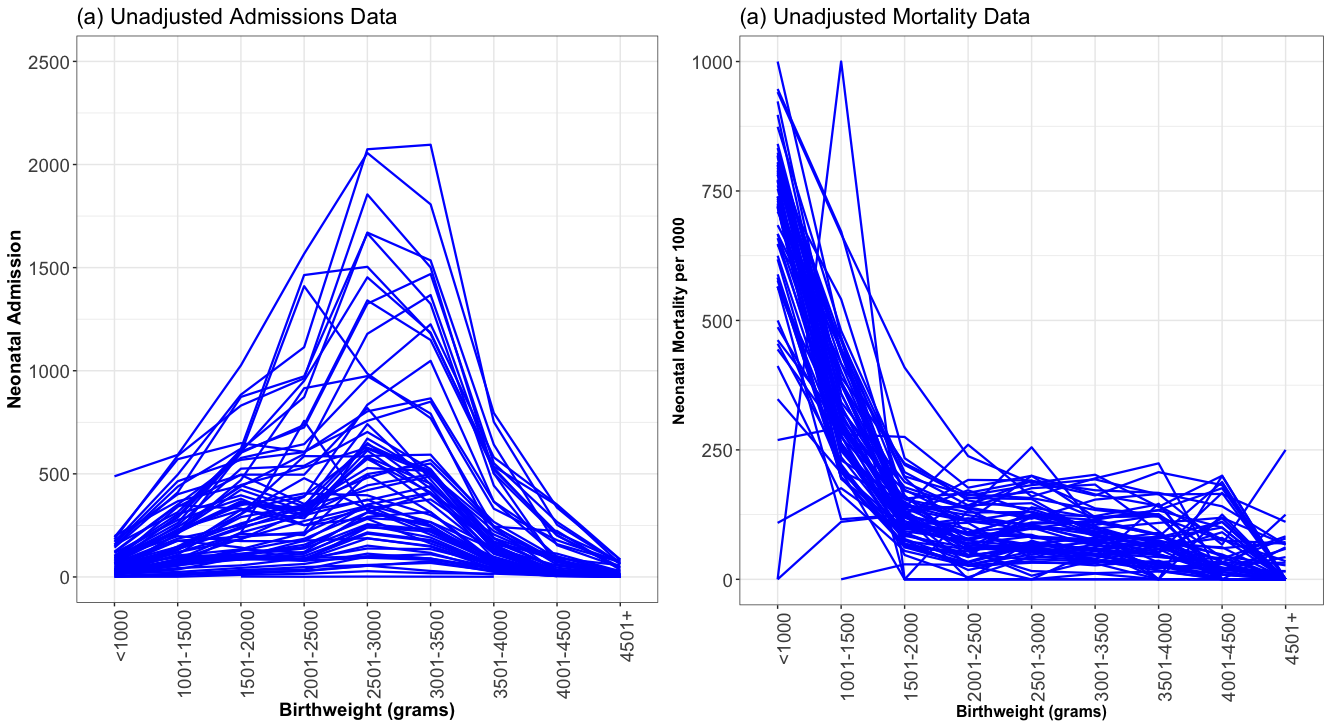


**eFigure 1 a**: Unadjusted Admissions and mortality data

**eFigure 1 b**: Unadjusted Admissions data

**eFigure 1 c**: Unadjusted NMR data

**Step 2**: Developing birthweight specific admission and NMR adjustment functions using NEST360 facilities with more plausible data

The results from the exploratory analysis in Step 1 indicated the presence of underreporting in the NEST360 baseline mortality dataset, particularly for the lowest birthweight categories. To address this issue, a subset of 34 neonatal units with plausible data was chosen for the development of admission and neonatal mortality rate (NMR) adjustment functions. The selection criteria for these neonatal units considered a threshold of at least 700 deaths per 1000 live births for babies with a birthweight less than 1000 grams. eFigure 1 d shows the distribution of the selected 34 neonatal units meeting this conservative threshold. This cutoff was considered conservative due to existing knowledge that the mortality rate in this group, in the absence of full intensive care, is no less than 800 per 1000 [1, 2].


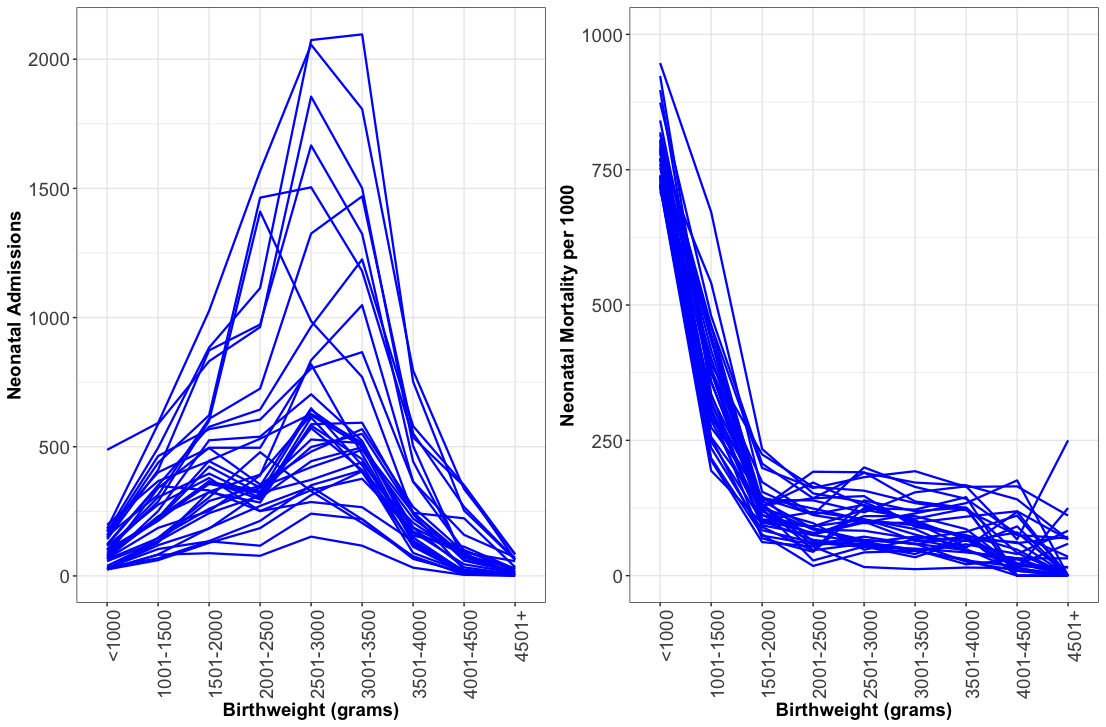


**eFigure 1 d**: BWS admissions and mortality curves for selected 34 facilities

*Adjustment curves for birthweight specific admissions*

For the birth weight (BWT) category *i*, the admissions model was expressed as:

${Admission}_{BWT=i}= \beta_{0}+ \sum_{j=1}^{8} \beta_{j}*{Admission}_{BWT\neq i}+ \epsilon$; where $\beta_{0}$ is the intercept with $\beta_{j}$ ( j = 1…8) representing parameters associated with admissions data in the remaining eight birthweight categories and $\epsilon$ the error term. In each of these models, the admission numbers were square root transformed [4] and modelled while assuming Gaussian distribution, with the resulting predictions back transformed to estimate ‘missing admitted’ babies.

*Adjustment functions for birthweight specific NMR*

In developing adjustment curves for mortality, regression models were specified separately for babies weighing less than 2500 grams and those weighing greater than or equal to 2500 grams, due to the acknowledged differences in risk levels between these two groups of babies [5]. The NMR models were therefore of the form:

${NMR}_{BWT=i}= \beta_{0}+ \sum_{j=1}^{n} \beta_{j}*{NMR}_{BWT\neq i}+ \epsilon$; where n is the number of birthweight categories; n = 4 and 5 for birthweight <2500g and ≥ 2500g respectively.

The unadjusted neonatal mortality rates (NMRs) were expressed as proportions, and a beta distribution with a logit link function was used in modelling NMR for each birth weight category. [6]. This was to restrict fitted values to the expected range between 0 and 1.

Bayesian modelling framework

All the admission and NMR models were fitted in a Bayesian framework to mitigate the potential impact of small sample size and data sparsity issues [7, 8]. For all the parameters, uniform priors were specified, and the models were fitted using three chains with 30,000 iterations. The first 50% of the iterations were designated as burn-in for each model. To assess the adequacy of the posterior distributions, history plots were used [9]. The convergence of the models was evaluated using the potential scale reduction factor (PSRF), which measures both the between- and within-chain variability in the sampled parameter values. An PSRF value of 1 indicates adequate convergence [10]. All the models converged as the reported PSRF for all the parameters were 1.

*Assessment of goodness of fit*

The goodness of fit for the adjustment functions was evaluated by comparing the observed and predicted data for the 34 selected neonatal units, and all were considered satisfactory as there were substantial overlaps between observed (dots) and fitted (blue lines) both for admissions and mortality data (eFigures 2 a and b).


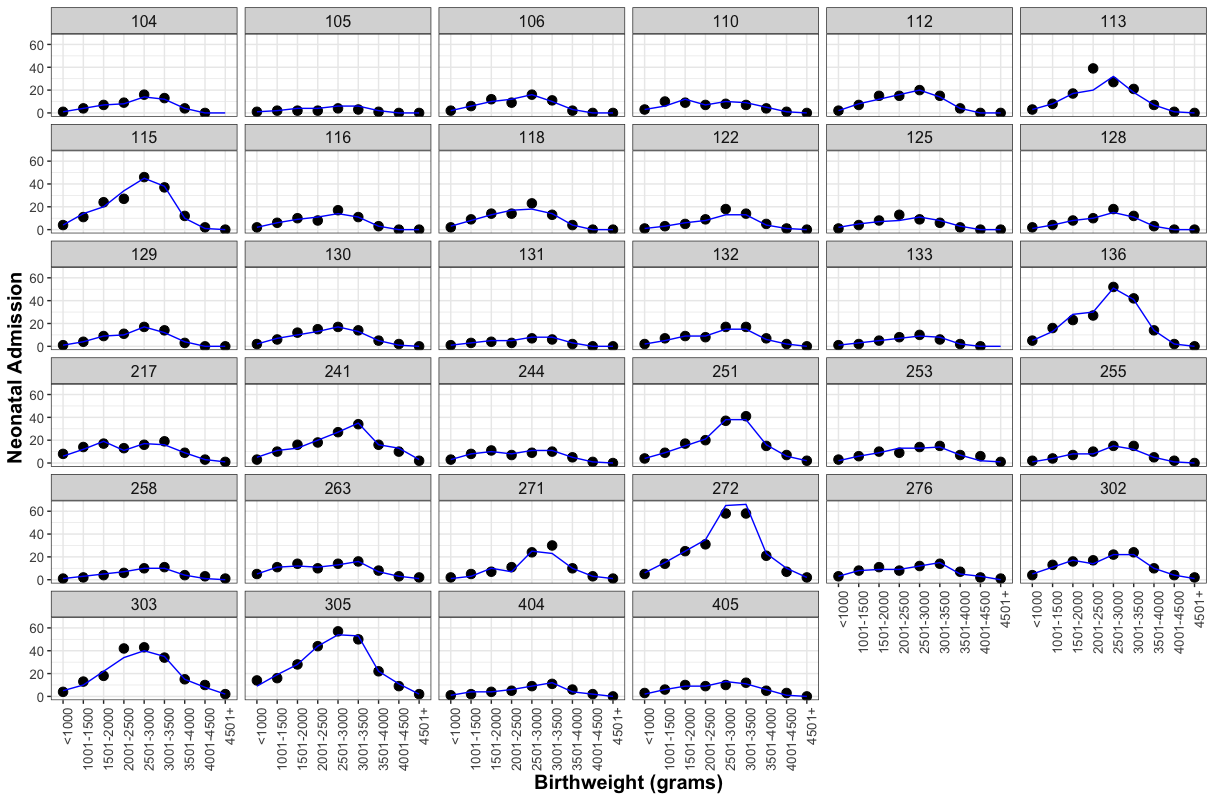


eFigure 2 a: Model fit for adjustment functions for admissions. Admission curves were based on monthly average admissions for each facility. Functions based on pooled mortality data (Jan 2019 to Dec 2021)


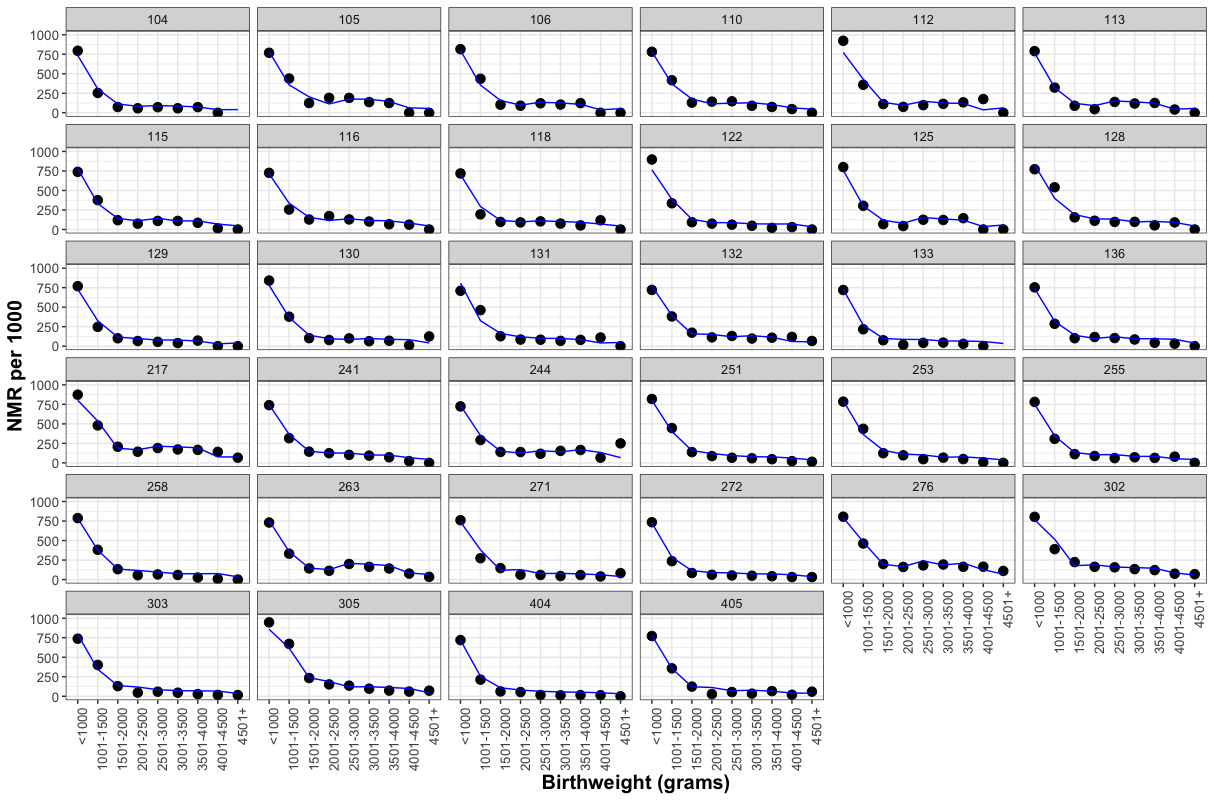


eFigure 2 b: Model fit for adjustment functions for NMR. Functions based on pooled mortality data (Jan 2019 to Dec 2021)

**Step 4: Applying the estimated adjustment functions to all NEST360 facilities to estimate adjusted admission and NMR trends**

The estimated adjustment functions were then used to adjust for underreporting and data gap in the rest of the facilities data. eFigures 3 a and b represent the adjusted and three monthly moving average smoothed trends for admissions and mortality data.

eFigure 3 a: Adjusted Admissions data

eFigure 3 b: Adjusted NMR data

**Comparison of unstructured and autoregressive variance covariance structures**

There were no discernable patterns (and mostly non-linear) in admission and mortality trends over time and therefore we assumed an unstructured variance-covariance structure for the mixed effects model which accounts for co-variances and autocorrelations, within the data. The mixed effects model incorporated four random effects: random intercept, random slope, step change, and slope change. The unstructured covariance structure effectively captured all variations arising from these random effects.

We re-analysed the data using an autoregressive covariance structure and compared to an unstructured covariance structure using the lower Akaike Information Criteria (AIC). eTable 1 below shows that an unstructured covariance structure was better as shown by lower AIC values.

**eTable 1**: Comparison of the performance of unstructured and autoregressive covariance structures

| Model (March interruption) | AIC (unstructured) | AIC (AR) |
| --- | --- | --- |
| Admissions model | **19994** | 20822 |
| NMR model | **-8085** | -7445 |

**Additional data for March and April 2020 interruption time points**

**Fit of the models**

eFigures 4 a and b show good and comparable fit for March and April 2020 interruption models, and this is demonstrated using data for selected facilities. Likelihood ratio test confirmed the need to account for non – linear trends using harmonic terms in both admissions and NMR models.

eFigure 4 a: Admissions model fit for March and April 2020 interruption time points for selected facilities

eFigure 4 b: Admissions model fit for March and April 2020 interruption time points for selected facilities

**Fitted Admissions and Mortality Trends for March 2020 interruption**

eFigure 5 a: Fitted Admissions (March 2020 Interruption)

eFigure 5 b: Fitted NMR (March 2020 Interruption)

**Fitted Admissions and Mortality Trends for April 2020 interruption**

eFigure 6 a: Fitted Admissions (April 2020 Interruption)

eFigure 6 b: Fitted NMR (April 2020 Interruption)

**eFigure 7**: Facility level step and slope change estimates for April 2020 interruption time-point

**eTable 2**: Model estimates for Time – Index Interruption Model

|  | **Admissions: log Rate Ratios** | | | **NMR: log Odds Ratios** | | |
| --- | --- | --- | --- | --- | --- | --- |
|  | Estimate | LCI | UCI | Estimate | LCI | UCI |
| (Intercept) | 5.0032 | 4.7274 | 5.279 | -1.5226 | -1.6901 | -1.3551 |
| Time | 0.0064 | 0.0016 | 0.0111 | 0.0041 | -0.0011 | 0.0094 |
| Index | -3.00E-04 | -0.0018 | 0.0012 | -7.00E-04 | -0.0026 | 0.0011 |
| Time:Index | **-1.00E-04** | **-2.00E-04** | **-0.000** | 0.000 | -1.00E-04 | 1.00E-04 |

NOTE: The segmented models adjusted for country as a fixed effect variable as well as Fourier terms to account for non-linearity in the trends

**Fitted Admissions and Mortality Trends for continuous OSI exposure**

eFigure 8 a: Fitted Admissions (Index – Time Interaction)

eFigure 8 b: Fitted Admissions (Index – Time Interaction)

LCI = Lower 95% CI; UCI – Upper 95% CI

**Stratified analysis by country**

We re-analysed the data separately for each country. In the country-specific models, 29 out of 67 facilities showed either borderline or significant reductions in admission numbers compared to 34 out of 67 we obtained in the pooled analysis from all countries (see Table 1 and 2 below). The difference of seven facilities were mostly in Malawi (6 of the 7 facilities) and 1 in Tanzania. The 6 facilities in Malawi are small facilities and hence the differences are due to small sample size.

**eTable 3**: Number of facilities with borderline or significant step reduction in admissions

| **Country** | **Mixed effects model** | **Country specific model** |
| --- | --- | --- |
| Tanzania | 5/7 | 5/7 |
| Kenya | 8/13 | 9/13 |
| Nigeria | 3/11 | 3/11 |
| Malawi | 18/36 | 12/36 |
| **Total** | **34/67** | **29/67** |

**eTable 4**: Number of facilities with borderline or significant month-to-month reduction in admissions

| **Country** | **Mixed effects model** | **Country specific model** |
| --- | --- | --- |
| Tanzania | 2/7 | 2/7 |
| Kenya | 1/13 | 2/13 |
| Nigeria | 0/11 | 2/11 |
| Malawi | 12/36 | 8/36 |
| **Total** | **15/67** | **14/67** |

**Changes in admission and referral patterns**

eFigure 9a: Admission trends by primary diagnosis

eFigure 9b: Admission trends by inborn/outborn

**References**

1. Blencowe, H., et al., *National, regional, and worldwide estimates of preterm birth rates in the year 2010 with time trends since 1990 for selected countries: a systematic analysis and implications.* (1474-547X (Electronic)).

2. Carlo, W.A., et al., *Newborn-care training and perinatal mortality in developing countries.* (1533-4406 (Electronic)).

3. McCarthy Bj Fau - Terry, J., et al., *The underregistration of neonatal deaths: Georgia 1974--77.* (0090-0036 (Print)).

4. St-Pierre, A.P., V. Shikon, and D.C. Schneider, *Count data in biology—Data transformation or model reformation?* Ecology and Evolution, 2018. **8**(6): p. 3077-3085.

5. Tomashek, K.M., et al., *Differences in Mortality between Late-Preterm and Term Singleton Infants in the United States, 1995&#x2013;2002.* The Journal of Pediatrics, 2007. **151**(5): p. 450-456.e1.

6. Ferrari, S. and F. Cribari-Neto, *Beta Regression for Modelling Rates and Proportions.* Journal of Applied Statistics, 2004. **31**(7): p. 799-815.

7. Greenland, S., M.A. Mansournia, and D.G. Altman, *Sparse data bias*

*a problem hiding in plain sight.* BMJ: British Medical Journal, 2016. **352**.

8. van de Schoot, R., et al., *Analyzing small data sets using Bayesian estimation: the case of posttraumatic stress symptoms following mechanical ventilation in burn survivors.* (2000-8066 (Print)).

9. Hamra, G., R. MacLehose, and D. Richardson, *Markov chain Monte Carlo: an introduction for epidemiologists.* International journal of epidemiology, 2013. **42**(2): p. 627-634.

10. Brooks, S.P. and A. Gelman, *General Methods for Monitoring Convergence of Iterative Simulations.* Journal of Computational and Graphical Statistics, 1998. **7**(4): p. 434-455.
