# Supplementary material for: COVID-19 pandemic effects on neonatal inpatient admissions and mortality: interrupted time series analysis of facilities implementing NEST360 in Kenya, Malawi, Nigeria, and Tanzania
Source: BMC Pediatr. 2024 Jul 8;23(Suppl 2):657. doi: 10.1186/s12887-024-04873-1 (PMC11232189; doi:10.1186/s12887-024-04873-1)
Supplement: Supplementary file 2 — Additional file 2. Ethics approval of Institutional Review Boards. [file 12887_2024_4873_MOESM2_ESM.docx]

**Additional file 2**: Ethics approval of Institutional Review Boards.

| **Institutional Review Boards** | **Date Granted** | **Number/REF** |
| --- | --- | --- |
| United Kingdom: London School of Hygiene & Tropical Medicine (LSHTM) Research Ethics Committee | 21/09/2020 | 21892 |
| Malawi: National Health Sciences Research Committee (NHSRC) | 10/01/2020 | NHSRC 1180 |
| Kenya: Kenya Medical Research Institute (KEMRI) | 27/05/2021 | KEMRI/SERU/CGMR-C/229/4203 |
| Kenya: Maseno University Ethics Review Committee | 24/02/2020 | MSU/DRPI/MUERC/00810/19 |
| Tanzania: Ifakara Health Institute (IHI) | 14/01/2020 | IHI/IRB/No: 01-2020 |
| Tanzania: National Institute for Medical Research (NIMRI) | 20/04/2020 | NIMR/HQ/R.8a/Vol. IX/3405 |
| Nigeria: Lagos University Teaching Hospital (LUTH) Health Research Ethics Committee | 10/02/2020 | ADM/DCST/HREC/APP/3487 |
| Nigeria: University of Ibadan/University College Hospital (UI/UCH) Ethics Committee | 13/05/2020 | NHREC/05/01/2008a |
